# Mediation analysis to understand genetic relationships between habitual coffee intake and gout

**DOI:** 10.1186/s13075-018-1629-5

**Published:** 2018-07-05

**Authors:** Joseph Hutton, Tahzeeb Fatima, Tanya J. Major, Ruth Topless, Lisa K. Stamp, Tony R. Merriman, Nicola Dalbeth

**Affiliations:** 1 Department of Medicine, Faculty of Medical and Health Sciences, University of Auckland, 85 Park Rd, Grafton, Auckland, New Zealand; 2 Department of Biochemistry, University of Otago, Dunedin, New Zealand; 3 Department of Medicine, University of Otago, Christchurch, New Zealand

**Keywords:** Gout, Coffee, Genetics, Diet, Urate

## Abstract

**Background:**

Increased coffee intake is associated with reduced serum urate concentrations and lower risk of gout. Specific alleles of the *GCKR*, *ABCG2*, *MLXIPL*, and *CYP1A2* genes have been associated with both reduced coffee intake and increased serum urate in separate genome-wide association studies (GWAS). The aim of this study was to determine whether these single nucleotide polymorphisms (SNPs) influence the risk of gout through their effects on coffee consumption.

**Methods:**

This research was conducted using the UK Biobank Resource. Data were available for 130,966 European participants aged 40–69 years. Gout status and coffee intake were tested for association with four urate-associated SNPs: *GCKR* (*rs1260326*), *ABCG2* (*rs2231142*), *MLXIPL* (*rs1178977*), and *CYP1A2* (*rs2472297*). Multiple regression and path analysis were used to examine whether coffee consumption mediated the effect of the SNPs on gout risk.

**Results:**

Coffee consumption was inversely associated with gout (multivariate adjusted odds ratio (95% confidence interval (CI)) for any coffee consumption 0.75 (0.67–0.84, *P =* 9 × 10^−7^)). There was also evidence of a dose-effect with multivariate adjusted odds ratio (95% CI) per cup consumed per day of 0.85 (0.82–0.87, *P =* 9 × 10^−32^). The urate-increasing *GCKR*, *ABCG2*, *MLXIPL*, and *CYP1A2* alleles were associated with reduced daily coffee consumption, with the strongest associations for *CYP1A2* (beta −0.30, *P =* 8 × 10^−40^), and *MLXIPL* (beta −0.17, *P =* 3 × 10^−8^), and weaker associations for *GCKR* (beta −0.07, *P =* 3 × 10^−10^) and *ABCG2* (beta −0.09, *P =* 2 × 10^−9^). The urate-increasing *GCKR* and *ABCG2* alleles were associated with gout (multivariate adjusted *p* < 5 × 10^−8^ for both), but the urate-increasing *MLXIPL* and *CYP1A2* alleles were not. In mediation analysis, the direct effects of *GCKR* and *ABCG2* accounted for most of the total effect on gout risk, with much smaller indirect effects mediated by coffee consumption.

**Conclusion:**

Coffee consumption is inversely associated with risk of gout. Although alleles at several SNPs associate with both lower coffee consumption and higher risk of gout, these SNPs largely influence gout risk directly, rather than indirectly through effects on coffee consumption.

**Electronic supplementary material:**

The online version of this article (10.1186/s13075-018-1629-5) contains supplementary material, which is available to authorized users.

## Background

Gout is a common inflammatory arthritis characterised by deposition of monosodium urate (MSU) crystals in joints and other tissues [1]. Hyperuricaemia is a key checkpoint in MSU crystallisation and the clinical presentation of gout [1]. Previous genome-wide association studies (GWAS) have identified 28 single nucleotide polymorphisms (SNPs) associated with serum urate concentration [2]. Some of these SNPs encode renal and/or gut urate transport-related proteins and associate with the risk of gout [2–5]. Others contribute to hyperuricaemia via regulation of purine synthesis and glucose metabolism pathways [2].

Long-established dietary factors for hyperuricaemia and gout risk include red meat, seafood, and alcohol [6, 7]. In addition, multiple studies have reported that increased coffee intake is associated with reduced serum urate [8–12] and risk of developing gout [13, 14]. This is the case for both caffeinated and de-caffeinated coffee. This association has been attributed to several potential mechanisms including improved insulin resistance [15–21] and caffeine-mediated inhibition of xanthine oxidase [8].

SNPs associated with habitual coffee consumption have also been identified by GWAS [22]. Of note, the GWAS of coffee consumption identified a number of genes which overlap with the genes identified in the separate hyperuricaemia GWAS [2, 22]. Specifically, alleles in several genes associated with hyperuricaemia (*GCKR*, *ABCG2*, *MLXIPL*, and *CYP1A2* [2]) are also associated with decreased habitual coffee intake [22]. These four alleles are the sole alleles currently known to be associated with both serum urate, gout risk, and habitual coffee consumption from existing genome-wide analysis studies. Regional association plots suggest that the signals for both urate and habitual coffee consumption are very similar for the four loci (Additional file 1: Figure S1). The aim of this study was to determine whether the lead urate-associated SNPs at the four coffee and serum urate associated loci influence the risk of gout through their effects on coffee consumption.

## Methods

### Participants

This research was conducted using the UK Biobank Resource [23, 24] (approval number 12611). Data for the UK Biobank were gathered over 2006–2010 from people between the ages of 40 and 69 years old. The North West Multi-Centre Research Ethics Committee granted ethical approval for UK Biobank participants. All participants provided written informed consent.

Inclusion criteria for this analysis were European ethnicity (white, British, Irish, any other white background) and genome-wide genotypes available. Exclusion criteria were self-reported sex mismatch with genetic sex, genotyping quality-control failure, and related individuals. Gout cases were ascertained in the UK Biobank using a validated case-definition of “self-reported gout or urate-lowering therapy (ULT) use” [25].

### Genotyping and SNP selection

UK Biobank samples were genotyped using an Affymetrix Axiom array (820,967 markers) and imputed to ~ 73.3 M SNPs using SHAPEIT3 and IMPUTE2, with a combined UK10K and 1000 Genomes reference panel.

Details of the lead coffee-associated SNP at each locus reported by Cornelis et al. (2015) [22] were extracted from the publication. The equivalent information was extracted from the serum urate GWAS publication for overlapping loci [2] and aligned to that of the coffee GWAS. For both *GCKR* and *CYP1A2*, the lead SNP reported in both studies was identical. For *ABCG2* and *MLIXPL*, the linkage disequilibrium (LD) between the lead urate-associated and coffee-associated SNP was calculated using the European 1000 Genomes phase 3 (September 2014) and UK Biobank data with PLINK v1.90 [26, 27]. The *ABCG2* SNPs were in high LD (*r*^2^_1000Genomes_ = 0.94; *r*^2^_UKBiobank_ = 0.99), whilst the *MLIXPL* SNPs were in moderate LD (*r*^2^_1000Genomes_ = 0.58; *r*^2^_UKBiobank_ = 0.57) (Table 1). Because of the adequate LD, the four urate-associated SNPs, *GCKR* (*rs1260326*), *ABCG2* (*rs2231142*), *MLXIPL* (*rs1178977*), and *CYP1A2* (*rs2472297*), were employed in the analysis (Table 1).

**Table 1:** SNPs associated with serum urate levels and habitual coffee intake in previous GWAS using participants of European ancestry

		*GCKR*	*ABCG2*	*MLXIPL*	*CYP1A2*
Coffee GWAS [20]	SNP	rs1260326	rs1481012	rs7800944	rs2472297
	Chr: Position (B37)	2:27730940	4:89039082	7:73035857	15:75027880
	Effect allele/other	T/C	A/G	T/C	T/C
	Effect allele freq.	0.41	0.89	0.72	0.24
	Beta (cups/day)	−0.04	0.06	−0.05	0.15
	SE	0.01	0.01	0.01	0.01
	*P*	1.06 × 10^−7^	1.13 × 10^−6^	7.82 × 10^−9^	6.45 × 10^−47^
Urate GWAS [2]	SNP	rs1260326	rs2231142	rs1178977	rs2472297
	Chr: Position (B37)	2:27730940	4:89271347	7:72494985	15:75027880
	Effect allele/other	T/C	G/T	A/G	T/C
	Effect allele freq.	0.41	0.89	0.81	0.24
	Beta (mg/dl)	0.07	−0.22	0.05	−0.03
	SE	0.01	0.01	0.01	0.01
	*P*	1.20 × 10^−44^	1.00 × 10^−134^	1.20 × 10^−12^	3.85 × 10^−6^
LD	Euro *r*^2^	Same SNP	0.94	0.58	Same SNP
			rs2231142: T	rs1178977: G	
			rs1481012: G	rs7800944: C	
	UKBB *r*^2^	Same SNP	0.99	0.57	Same SNP
			rs2231142: T	rs1178977: G	
			rs1481012: G	rs7800944: C	

Linkage disequilibrium calculated using 1000 Genomes phase 3 (September 2014) data

*Chr* chromosome, *Euro* European from 1000 Genomes, *Freq* frequency, *GWAS* genome-wise association study, *LD* linkage disequilibrium, *SE* standard error, *SNP* single nucleotide polymorphism, *UKBB* UK Biobank

### Coffee and food-frequency intake

At the time of recruitment, data were collected on usual coffee consumption in addition to other foodstuffs [28]. Coffee intake was determined by participants’ answers to the question “How many cups of coffee do you drink each day (include de-caffeinated coffee)?” Values were numerical in exact cups per day (including 0) or under 1 cup per day. Any non-zero value was determined as “any” coffee consumption. Other food intakes were determined by responses to questions about daily or weekly food frequency [28] (Additional file 2: Table S1 for codes).

### Statistical analysis

Multivariate linear and logistic regression analysis was completed using SPSS version 24 (IBM, New York, USA). All models were adjusted for age, sex, body mass index (BMI), hypertension, kidney disease, diabetes mellitus, and reported intake of cups of tea, fruit, vegetables, meat, fish, bread, cereal, and cheese. Beer and spirits were included as additional variables in separate analyses due to high levels of missing data, with only 70.4% of participants having information on beer/cider intake and 70.2% of participants with information on spirit intake. The effect of each SNP was analysed using two models, a dominant model (for the presence of least one urate-raising allele) and a recessive model (homozygosity for the urate-raising allele). Experiment-wide significance was set at *P* < 0.0125 after Bonferroni correction for multiple testing (0.05 divided by four SNPs). For gout, multivariate logistic regression analysis with gout as the dependent variable and coffee intake or SNP status as the independent variables was completed. For coffee intake, multivariate linear regression analysis was completed with coffee intake (cups per day) as the dependent variable and SNP status as the independent variables. All regression analyses were fully adjusted as described above.

The PROCESS macro v2.16.3 for SPSS was used to construct a mediation pathway to gout risk. Model 4 (used for simple mediation models exploring the relationship between a single independent variable, single mediator, and single dependent variable as presented in Additional file 3: Figure S2) was used from the PROCESS macro with 1000 bootstraps. This aimed to quantify the direct and indirect relationships between SNPs, coffee intake, and gout. Only SNPs associated with both gout and coffee consumption in the UK Biobank data (*GCKR* and *ABCG2*) were included in the mediation analysis; this aimed to establish whether the effects of *GCKR*/*ABCG2* on gout risk were mediated through coffee intake. Direct and indirect standardised effect estimates were calculated using multivariate-adjusted linear and logistic regression analysis as described above. All effect estimates were adjusted for the same potential confounding variables as described above. Bootstrapping was used to determine whether the indirect effect of the SNP on gout risk through coffee intake was significant.

## Results

### Participant demographics

Data including dietary information and genome-wide genotypes were available for 130,966 participants, including 2135 participants with gout. The demographic and clinical information of participants is shown in Table 2, with full dietary information shown in Additional file 4 (Table S2). Participants with gout were older, more likely to be male, had higher mean BMI, and had a higher prevalence of co-morbid conditions.

**Table 2:** Demographic and clinical characteristics of study population (*n* = 130,966)

	Controls	% with data available	Gout cases	% with data available
*n*	128,831		2135	
Males, *n* (%)	59,782 (46.4%)	100.0	1972 (92.4%)	100.0
Age (years)	56.7 (7.98)	100.0	60.23 (6.67)	100.0
Body mass index (kg /m^2^)	27.47 (4.82)	99.7	30.87 (5.01)	99.6
Townsend Index	−1.38 (3.05)	99.9	−1.12 (3.10)	99.8
Hypertension, *n* (%))	40,185 (31.2%)	100.0	1402 (65.7%)	100,0
Diabetes, *n* (%)	5384 (4.2%)	100.0	279 (13.1%)	100.0
Kidney disease, *n* (%)	1121 (0.9%)	100.0	125 (5.9%)	100.0
Meat consumption, *n* (%)	122,658 (95.2%)	98.9	2103 (98.5%)	99.2
All meat intake (pieces per week)	5.50 (2.72)	98.9	6.55 (2.77)	99.2
Fish consumption, *n* (%)	123,330 (95.7%)	99.3	2079 (97.4%)	99.2
All fish intake (pieces per week)	2.23 (1.58)	99.3	2.34 (1.63)	99.2
Beer/cider intake (pints per week)	3.11 (5.71)	70.2	9.11 (10.26)	83.8
Spirits intake (measures per week)	1.98 (5.91)	70.0	2.97 (8.99)	83.4
Coffee consumption, *n* (%)	101,076 (78.5%)	99.8	1617 (75.7%)	99.2
Coffee intake (cups per day)	2.12 (2.20)	99.8	1.75 (1.83)	99.2
Tea consumption, *n* (%)	108,734 (84.4%)	99.8	1813 (84.9%)	99.9
Tea intake (cups per day)	3.45 (2.99)	99.8	3.31 (2.88)	99.9
Any fruit consumption, *n* (%)	119,605 (92.8%)	98.9	1917 (89.8%)	98.0
Fruit of any description (pieces per day)	2.98 (2.50)	98.9	2.72 (2.33)	98.0
All vegetable intake (pieces per day)	4.83 (3.17)	98.6	4.83 (3.06)	97.3
Bread intake (slices per week)	12.52 (8.69)	99.2	14.77 (9.72)	98.7
Cereal intake (bowls per week)	4.52 (2.80)	99.7	3.83 (2.81)	99.7
Cheese intake (pieces per week)	2.46 (1.75)	97.7	2.35 (1.66)	96.9

Hypertension, diabetes mellitus, and kidney disease defined by self-reported illness or hospital diagnosis

All values are shown as mean (standard deviation) unless otherwise indicated

### Coffee consumption and gout

Coffee consumption was inversely associated with gout (Table 3). Multivariate adjusted odds ratio (OR) (95% confidence interval (CI)) of gout for any coffee consumption was 0.75 (0.67–0.84, *P =* 9 × 10^−7^) and 0.85 (0.82–0.87, *P =* 9 × 10^−32^) per cup of coffee consumed per day. These associations persisted after intake of beer and spirits was added to the models.

**Table 3:** Association of coffee intake with gout

		Association of coffee intake (any coffee) with gout		
	Observations	Odds ratio	95% confidence interval	*P*
Unadjusted	130,731	0.85	0.77–0.94	0.002
Adjusted^†^	121,897	0.75	0.67–0.84	9.05 × 10^−7^
Adjusted^†^ including beer/spirits	86,676	0.75	0.66–0.86	1.40 × 10^−5^
		Association of coffee intake (per cup per day) with gout		
	Observations	Odds ratio	95% confidence Interval	*P*
Unadjusted	130,731	0.91	0.89–0.93	3.08 × 10^−15^
Adjusted^†^	121,897	0.85	0.82–0.87	8.55 × 10^−32^
Adjusted^†^ including beer/spirits	86,676	0.85	0.83–0.88	1.09 × 10^−23^

^†^Adjusted for age, sex, body mass index, hypertension, kidney disease, diabetes, meat intake, fish intake, cheese intake, tea intake, fruit intake, vegetable intake, bread intake, and cereal intake

### Association of SNPs with gout risk

The urate-associated SNPs for both *GCKR* and *ABCG2* were significantly associated with gout (Table 4). The strongest observed association was for *ABCG2* with multivariate adjusted OR 2.37 (95% CI 2.15–2.61, *P =* 6 × 10^−69^). *GCKR* also had a strong effect on gout risk with multivariate adjusted OR 1.43 (95% CI 1.29–1.58, *P =* 4 × 10^−12^). Neither *MLXIPL* nor *CYP1A2* were associated with gout, with multivariate adjusted OR 1.24 (95% CI 0.95–1.61, *P =* 0.11) and 1.16 (95% CI 0.96–1.40, *P =* 0.12), respectively. For *GCKR* and *ABCG2*, association with gout was observed for both the presence of at least one urate-raising allele (Table 4) as well as by number of urate-raising alleles (Additional file 5: Table S3).

**Table 4:** Association analysis of urate-associated SNPs with gout and coffee intake

			Association of gout with urate-associated SNPs					Association of coffee intake (cups per day) with urate-associated SNPs				
Gene	SNP	Effect allele	Observations	%	Odds ratio	95% Confidence interval	*P*	Observations	%	Unadjusted β coefficient	95% Confidence interval	*P*
*GCKR*												
Unadjusted	rs1260326	T	130,966	100.0%	1.40	1.27–1.53	3.34 × 10^−12^	130,731	100.0%	−0.07	−0.10 to −0.05	1.33 × 10^−8^
Adjusted†			121,940	93.1%	1.43	1.29–1.58	4.05 × 10^−12^	121,897	93.2%	−0.08	−0.10 to −0.05	3.05 × 10^−10^
Adjusted† including beer/spirits			86,702	66.2%	1.45	1.30–1.62	2.32 × 10^−11^	86,676	66.3%	−0.07	−0.10 to −0.05	2.47 × 10^−8^
*ABCG2*												
Unadjusted	rs2231142	T	130,966	100.0%	2.26	2.07–2.47	1.05 × 10^−72^	130,731	100.0%	−0.07	−0.10 to −0.04	1.00 × 10^−6^
Adjusted†			121,940	93.1%	2.37	2.15–2.61	6.62 × 10^−69^	121,897	93.2%	−0.09	−0.11 to −0.06	2.10 × 10^−9^
Adjusted† including beer/spirits			86,702	66.2%	2.45	2.20–2.72	4.78 × 10^−61^	86,676	66.3%	−0.08	−0.11 to −0.05	3.99 × 10^−7^
*MLXIPL*												
Unadjusted	rs1178977	A	130,966	100.0%	1.25	0.98–1.61	0.07	130,702	100.0%	−0.15	−0.21 to −0.09	3.00 × 10^−6^
Adjusted^†^			121,940	93.1%	1.24	0.95–1.61	0.11	121,897	93.2%	−0.17	−0.23 to −0.11	3.20 × 10^−8^
Adjusted^†^ including beer/spirits			86,702	66.2%	1.24	0.94–1.65	0.13	86,661	66.3%	−0.21	−0.27 to −0.14	5.45 × 10^−10^
*CYP1A2*												
Unadjusted	rs2472297	C	130,966	100.0%	1.15	0.96–1.37	0.13	130,731	100.0%	−0.25	−0.30 to −0.21	3.02 × 10^−26^
Adjusted^†^			121,940	93.1%	1.16	0.96–1.40	0.12	121,897	93.2%	−0.30	−0.35 to −0.26	7.61 × 10^−40^
Adjusted^†^ including beer/spirits			86,702	66.2%	1.16	0.94–1.42	0.17	86,676	66.3%	−0.30	−0.35 to −0.25	2.80 × 10^−32^

Analysis is shown for the presence of at least one urate-raising allele

Effect allele is allele associated with hyperuricaemia in Kottgen GWAS paper [2]

*SNP* single nucleotide polymorphism

^†^Adjusted for age, sex, body mass index, hypertension, kidney disease, diabetes, meat intake, fish intake, cheese intake, tea intake, fruit intake, vegetable intake, bread intake, and cereal intake

### Association of SNPs with coffee intake

All four urate-associated SNPs were also associated with reduced coffee intake (Table 4). *CYP1A2* showed the strongest association with reduced coffee intake (beta −0.30, 95% CI −0.35 to −0.26, *P =* 8 × 10^−40^), followed by *MLXIPL* (beta −0.17, 95% CI −0.23 to −0.11, *P =* 3 × 10^−8^). Weaker associations were observed with *GCKR* (beta −0.08, 95% CI −0.11 to −0.06, *P =* 3 × 10^−10^) and *ABCG2* (beta −0.09, 95% CI −0.11 to −0.06, *P =* 2 × 10^−9^). All SNPs demonstrated evidence of a dose effect for coffee consumption with the strongest association evident in those with two copies of the urate-raising allele (Additional file 6: Table S4).

### Mediation analysis

In logistic regression models that included urate- and gout-associated SNPs, coffee consumption was inversely associated with gout (Table 5). In these models, the association of both *GCKR* and *ABCG2* with gout were also observed. Association of coffee consumption with gout was independent of all SNPs, and association of *GCKR* and *ABCG2* SNPs with gout was independent of coffee consumption.

**Table 5:** Logistic regression models including adjustment for both coffee intake and urate-associated SNPs for association with gout

Variable	Odds ratio for gout	95% Confidence Interval	Standard Error	P
*GCKR (*rs1260326)	1.42	1.28 - 1.57	0.05	7.78E^-12^
Coffee consumption (any)	0.76	0.68 - 0.85	0.06	2.00E^-06^
*ABCG2 (*rs2231142)	2.36	2.14 -2.60	0.05	2.07E^-68^
Coffee consumption (any)	0.76	0.68 - 0.85	0.06	2.00E^-06^
*MLXIPL (*rs1178977)	1.23	0.95 -1.60	0.13	0.12
Coffee consumption (any)	0.75	0.67 - 0.84	0.06	9.45E^-07^
*CYP1A2 (*rs2472297)	1.15	0.96 - 1.39	0.10	0.14
Coffee consumption (any)	0.75	0.67 - 0.84	0.06	1.00E^-06^
*GCKR (*rs1260326)	1.41	1.27 - 1.55	0.05	2.79E^-11^
Coffee intake (per cup per day)	0.85	0.83 - 0.87	0.01	4.29E^-31^
*ABCG2 (*rs2231142)	2.33	2.12 - 2.57	0.05	1.83E^-66^
Coffee intake (per cup per day)	0.85	0.83 - 0.88	0.01	4.02E^-30^
*MLXIPL (*rs1178977)	1.20	0.92 - 1.56	0.13	0.17
Coffee intake (per cup per day)	0.85	0.82 - 0.87	0.01	1.15E^-31^
*CYP1A2 (*rs2472297)	1.11	0.92 - 1.34	0.10	0.27
Coffee intake (per cup per day)	0.85	0.83 - 0.87	0.01	1.41E^-31^

Effect allele is allele associated with hyperuricaemia in Kottgen GWAS paper (2)

Odds are shown for presence/absence of urate-raising allele

All results shown are adjusted for age, sex, body mass index, hypertension, kidney disease, diabetes, meat intake, fish intake, cheese intake, tea intake, fruit intake, vegetable intake, bread intake, and cereal intake

*SNP* single nucleotide polymorphism

**Fig. 1:**
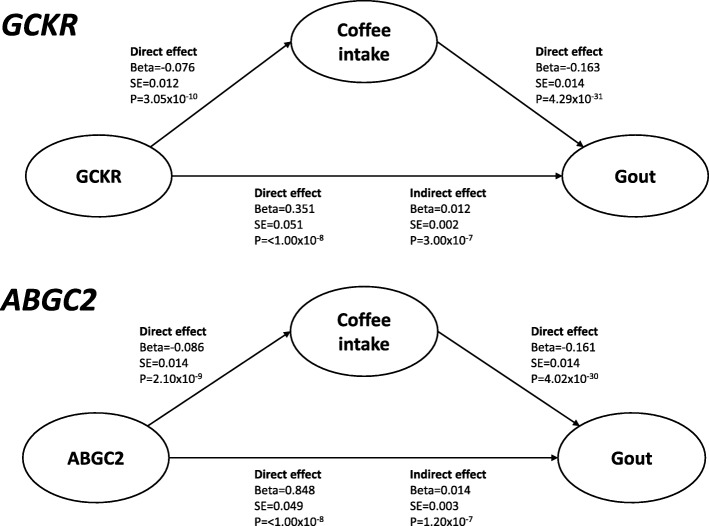
Summary of mediation analysis. Standardised path coefficients are shown. The direction of the path analysis from SNP to gout was pre-specified. Analysis is shown for the presence of at least one urate-raising allele and coffee intake in cups per day. Effect sizes are shown when results are adjusted for the following co-variates: age, sex, body mass index, hypertension, kidney disease, diabetes mellitus, and intake of meat, fish, cheese, tea, fruit, vegetables, bread, and cereal. SE standard error

Mediation results are shown in Fig. 1. In mediation analysis of *GCKR*, a strong direct effect of the urate-associated SNP on gout risk was demonstrated (beta 0.351, standard error (SE) 0.051, *P* < 1.00 × 10^−8^). A direct effect of the urate-associated SNP on coffee intake was also demonstrated (beta −0.076, SE = 0.012, *P* = 3.05 × 10^−10^). The direct effect of the urate-associated SNP accounted for most of the total observed effect on gout risk, with much smaller indirect effects mediated by coffee consumption (beta 0.012, SE 0.002 compared with beta 0.351, SE 0.051).

Similarly, in mediation analysis of *ABCG2*, a strong direct effect of the urate-associated SNP on gout risk was demonstrated (beta 0.848, SE 0.049, *P* < 1.00 × 10^−8^). A direct effect of the urate-associated SNP on coffee intake was also demonstrated (beta −0.086, SE 0.014, *P* = 2.10 × 10^−9^). The direct effect of the urate-associated *ABCG2* SNP accounted for most of the total observed effect on gout risk, with a much smaller indirect effect mediated by coffee consumption (beta 0.848, SE 0.049 compared with beta 0.014, SE 0.03).

## Discussion

This study provides further evidence that coffee intake is inversely associated with gout. This association was observed in those who consumed any coffee versus none, with evidence of a dose-effect response for the number of cups consumed per day. Although the urate-raising *GCKR* and *ABCG2* alleles were associated with both lower coffee consumption and higher risk of gout, mediation analysis demonstrated that these SNPs largely influence gout risk directly, rather than indirectly through their dual effect on coffee consumption.

In our analysis of the UK Biobank, all of the loci from previous GWAS of habitual coffee consumption [22] tested here (*ABCG2*, *GKCR*, *MLXIPL*, and *CYP1A2*) were replicated, strengthening evidence for the genetic basis of this trait. *CYP1A2* and its product, cytochrome P450, have been demonstrated to have a central role in the metabolism of caffeine [29]. Indeed, *CYP1A2* is one of the strongest known loci for coffee consumption, both in this report and in other studies [30]. Our results are consistent with a recent GWAS meta-analysis that reported genome-wide significant associations of the *CYP1A2* locus and nominal association of the *GCKR* and *ABCG2* loci with plasma caffeine levels [31].

Despite the association of *GCKR* and *ABCG2* with both gout and reduced coffee intake, our mediation analysis indicates that the dominant mechanism for *GCKR* and *ABCG2* on gout risk is not through coffee consumption. The urate-raising alleles of these SNPs are associated with reduced coffee intake; this suggests that implementing a dietary intervention such as increased coffee consumption may be more difficult in those genetically pre-disposed towards gout as urate levels would be raised. Analysis of modifiable exposures associated with gout risk alleles is an avenue for further study as GWAS for various dietary factors become more common. Indeed, a recent UK Biobank study identified several SNPs associated with alcohol consumption, including *GCKR rs1260326* [32]. This is especially interesting given the recently reported interaction between *GCKR rs780094* (in very strong linkage disequilibrium with *rs1260326*), alcohol, and gout risk [33].

We did not use Mendelian randomisation to directly address the question of the causality of coffee consumption in regulating serum urate levels and the risk of gout. This is because the majority of the coffee consumption-associated genetic variants also associate with serum urate levels making the variants unsuitable as Mendelian randomisation instrumental variables. However, we note that the *AHR* (aryl hydrocarbon receptor) locus, with an approximately equal effect size on coffee consumption as *CYP1A2* [22], does not associate with serum urate levels [2]. Consistent with our mediation analysis, this does not support a direct causal role of coffee consumption in regulating serum urate levels. Very recently, a conventional Mendelian randomisation study [34] provided support for a causal role of coffee consumption in reducing the risk of gout. However, the authors of this Mendelian randomisation also observed in the separate gout case-control data set [2] that genetic variation in *AHR,* one of the strongest genetic effects on habitual coffee consumption [22], did not contribute to the protective effect for gout. One of the fundamental assumptions of Mendelian randomisation is that the SNPs used for the instrumental variables are unrelated to the outcome [35]. Although statistical methods exist for evaluating the effect of pleiotropy in Mendelian randomisation studies, the fact that coffee- and urate-associated loci overlap limits the application of Mendelian randomisation to assessing a possible causal role of coffee in gout.

This study has several limitations. Only three of the four urate-associated SNPs used in this study were identical to (or in very high LD with) the SNPs reported in the previous coffee GWAS. However, we did observe an association between coffee consumption and all four urate-associated SNPs tested in this analysis. The study population is restricted to those of white European ancestry, thereby limiting the generalisability to other populations. There may also be some bias in reported dietary intakes. Furthermore, widely used food-frequency questionnaires lack specificity for some foodstuffs [36]. Any effect of this is likely minimised due to the UK Biobank’s extremely comprehensive data collection gathered through multiple modalities. Similarly, the non-specific nature of data collection for general use rather than specific research questions may also help minimise the effects of recall bias. Key strengths of this study include the large sample size and wide access to multiple data sources such as self-reported illness, medications, hospital records, and dietary data.

## Conclusions

In summary, this work further supports the hypothesis that coffee consumption is protective for gout risk. The exact mechanism of this protective effect remains unclear. Although several SNPs associate with both lower total coffee consumption and higher gout risk, mediation analysis indicates that these SNPs have direct effects on gout risk rather than indirect effects mediated by coffee consumption. The coffee- and urate-associated loci could influence coffee consumption and urate levels, respectively, through separate biological mechanisms.

## Additional files

Additional file 1: **Figure S1.** Regional association plots of genome-wide significant urate and habitual coffee loci. In each panel, SNPs identified as associated with both urate [2] and coffee intake [22] are plotted with their –log_10_ (*P* values) as a function of genomic position using HG build 19 and 1000 genomes European reference for LD (November 2014). Each SNP is coloured according to its correlation with the index SNP (demonstrating the lowest *P* value within the region, labelled in purple) according to a scale from *r*^2^ = 0 to *r*^2^ = 1. Urate-raising alleles are displayed on the left and coffee-associated alleles are on the right. LocusZoom plots were drawn from publicly available data ex [2] and taken from [22]. *GCKR rs2911711* is in complete linkage disequilibrium with *rs1260326.* (PDF 826 kb)

Additional file 2: **Table S1.** Coding of food-frequency intakes for analysis. (DOC 40 kb)

Additional file 3: **Figure S2.** Description of PROCESS model 4. (PDF 29 kb)

Additional file 4: **Table S2.** Complete dietary information for the study population. (DOCX 16 kb)

Additional file 5: **Table S3.** Association analysis of genotype with gout risk. (DOC 57 kb)

Additional file 6: **Table S4.** Association analysis of genotype with habitual coffee intake. (DOC 54 kb)

## Abbreviations

*ABCG2*: ATP-binding cassette subfamily G member 2
BMI: Body mass index
CI: Confidence interval
*CYP1A2*: Cytochrome P450 family 1 subfamily A member 2
*GCKR*: Glucokinase regulator
GWAS: Genome-wide association studies
LD: Linkage disequilibrium
*MLXIPL*: MAX-like protein X interacting protein like
MSU: Monosodium urate
OR: Odds ratio
SE: Standard error
SNP: Single nucleotide polymorphism
ULT: Urate-lowering therapy
