# Supplementary figures and images for: Mediation analysis to understand genetic relationships between habitual coffee intake and gout
Source: Arthritis Res Ther. 2018 Jul 5;20:135. doi: 10.1186/s13075-018-1629-5 (PMC6034252; doi:10.1186/s13075-018-1629-5)

**GCKR**

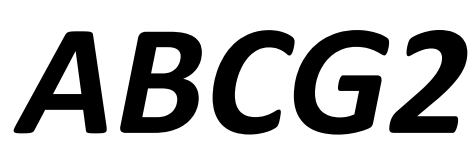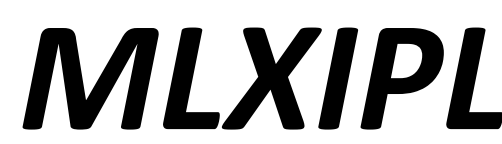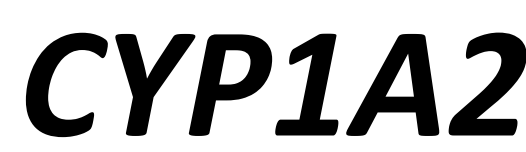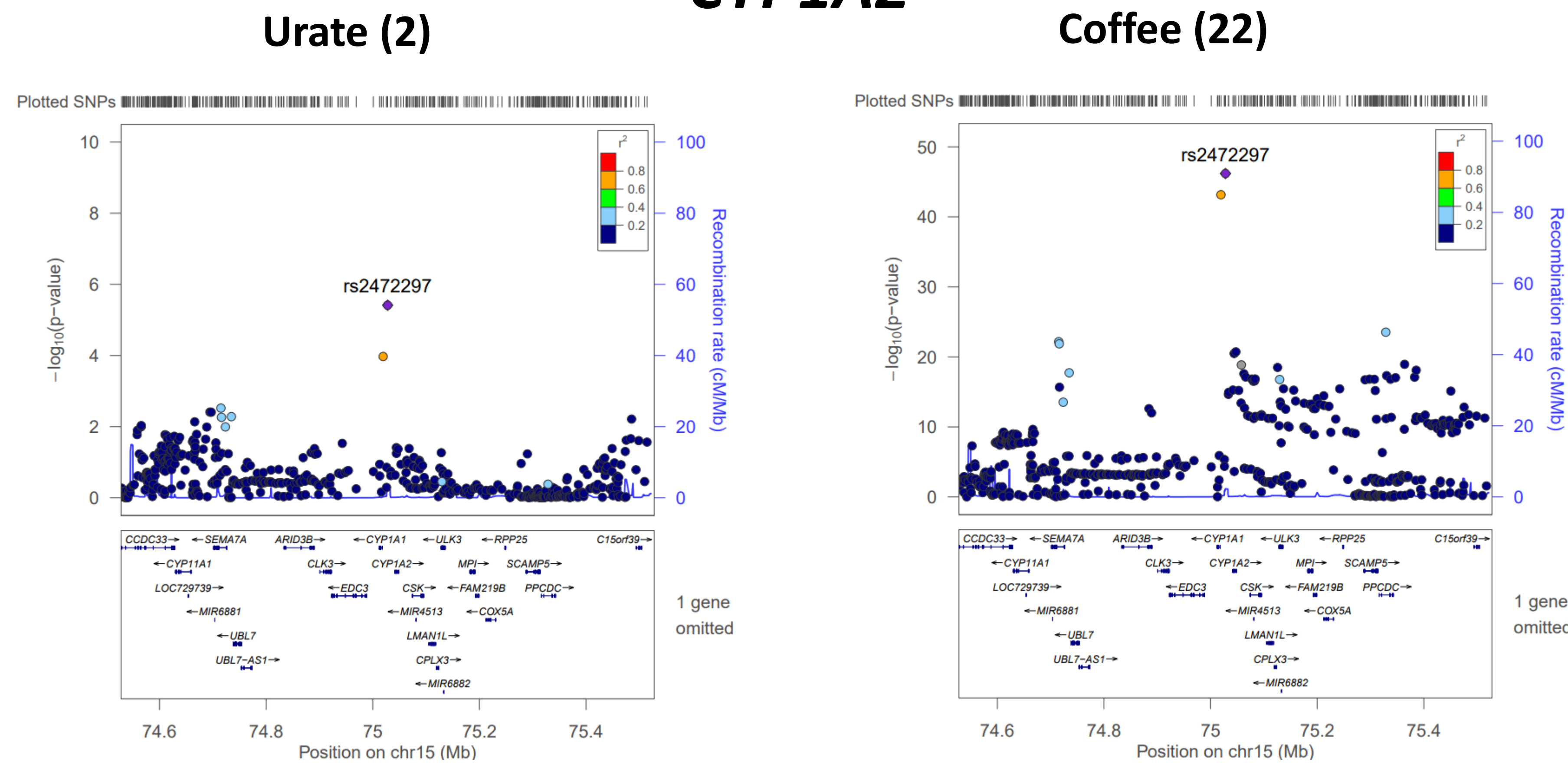

Supplement: Supplementary file 1 — Figure S1. Regional association plots of genome-wide significant urate and habitual coffee loci. In each panel, SNPs identified as associated with both urate [2] and coffee intake [22] are plotted with their –log10 (P values) as a function of genomic position using HG build 19 and 1000 genomes European reference for LD (November 2014). Each SNP is coloured according to its correlation with the index SNP (demonstrating the lowest P value within the region, labelled in purple) according to a scale from r2 = 0 to r2 = 1. Urate-raising alleles are displayed on the left and coffee-associated alleles are on the right. LocusZoom plots were drawn from publicly available data ex [2] and taken from [22]. GCKR rs2911711 is in complete linkage disequilibrium with rs1260326. (PDF 826 kb) [file 13075_2018_1629_MOESM1_ESM.pdf]

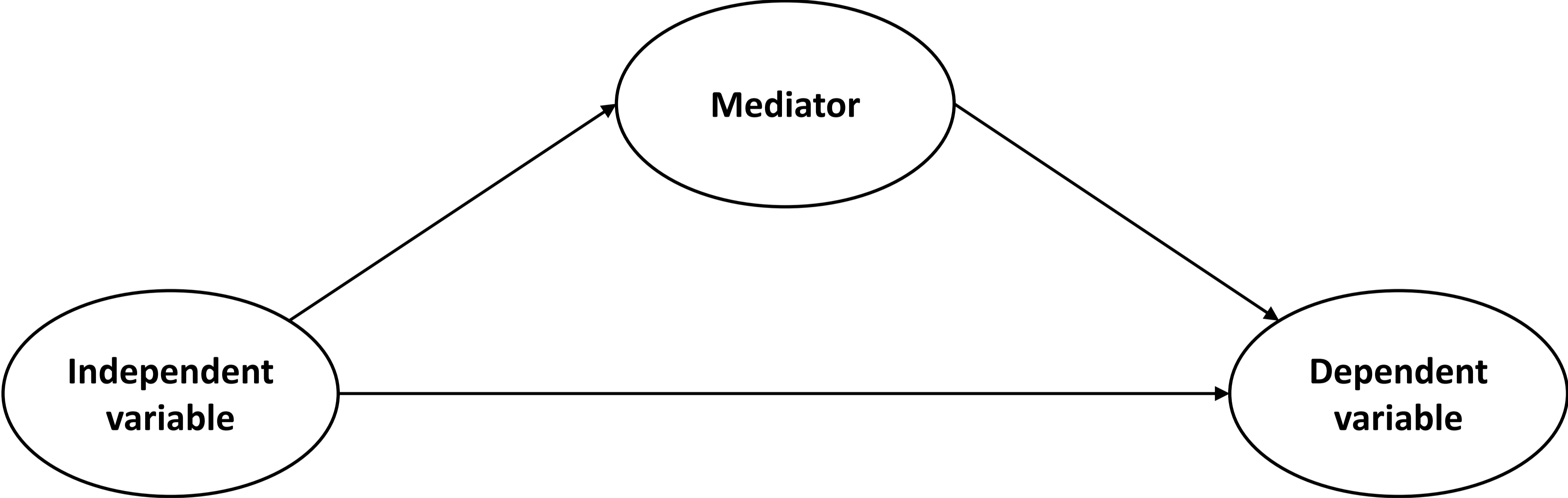

Supplement: Supplementary file 3 — Figure S2. Description of PROCESS model 4. (PDF 29 kb) [file 13075_2018_1629_MOESM3_ESM.pdf]
