# Supplementary material for: Mediation analysis to understand genetic relationships between habitual coffee intake and gout
Source: Arthritis Res Ther. 2018 Jul 5;20:135. doi: 10.1186/s13075-018-1629-5 (PMC6034252; doi:10.1186/s13075-018-1629-5)
Supplement: Supplementary file 2 — Table S1. Coding of food-frequency intakes for analysis. (DOC 40 kb) [file 13075_2018_1629_MOESM2_ESM.doc]

**Table S1. Coding of food-frequency intakes for analysis.**

| **UK Biobank coding†** | **Response category** | **Codes used for analysis (value/week)** |
| --- | --- | --- |
|  | **Meat / cheese / fish per week** |  |
| -3 and -1 | "Prefer not to answer" and "do not know" | Not included |
| 0 | Never | 0 |
| 1 | Less than once a week | 0.47 |
| 2 | Once a week | 1 |
| 3 | 2-4 times a week | 3 |
| 4 | 5-6 times a week | 5.5 |
| 5 | Once or more daily | 7 |
|  | **Slices of bread / bowls of cereal per week** |  |
| -3 and -1 | "Prefer not to answer" and "do not know" | Not included |
| 0 | None | 0 |
| -10 | Less than one | 3.5 |
| Numeric | Number of slices | Numeric |
|  | **Beer and spirits per week** |  |
| -3 and -1 | "Prefer not to answer" and "do not know" | Not included |
| 0 | Never | 0 |
| Numeric | Number of pints (or measures) drunk | Numeric |
| ***† Coding used within relevant Biobank field*** | |  |
