# Supplementary material for: Mediation analysis to understand genetic relationships between habitual coffee intake and gout
Source: Arthritis Res Ther. 2018 Jul 5;20:135. doi: 10.1186/s13075-018-1629-5 (PMC6034252; doi:10.1186/s13075-018-1629-5)
Supplement: Supplementary file 4 — Table S2. Complete dietary information for the study population. (DOCX 16 kb) [file 13075_2018_1629_MOESM4_ESM.docx]

**Table S2. Complete dietary information for the study population.** All values aside from n (%), data shown as mean (SD).

|  | **Control** | **% with data available** | **Gout** | **% with data available** |
| --- | --- | --- | --- | --- |
| Meat of any description consumption, n (%) | 122658 (95.2%) | 98.9 | 2103 (98.5%) | 99.2 |
| All meats intake (pieces per week) | 5.50 (2.72) | 98.9 | 6.55 (2.77) | 99.2 |
| Red meat consumption, n (%) | 121417 (94.3%) | 99.9 | 2099 (98.3%) | 99.2 |
| Red meat intake (pieces per week) | 3.59 (2.22) | 99.9 | 4.56 (2.43) | 99.2 |
| Processed meat intake (pieces per week) | 1.51 (1.41) | 99.9 | 2.04 (1.57) | 99.8 |
| Beef intake (pieces per week) | 0.90 (0.86) | 99.6 | 1.08 (0.95) | 99.7 |
| Lamb intake (pieces per week) | 0.57 (0.52) | 99.4 | 0.70 (0.60) | 99.6 |
| Pork intake (pieces per week) | 0.61 (0.57) | 99.5 | 0.75 (0.66) | 99.7 |
| Poultry intake (pieces per week) | 1.91 (1.24) | 99.8 | 1.99 (1.19) | 100.0 |
| Fish of any description consumption, n (%) | 123330 (95.7%) | 99.3 | 2079 (97.4%) | 99.2 |
| All fish intake (pieces per week) | 2.23 (1.58) | 99.3 | 2.34 (1.63) | 99.2 |
| Oily fish (pieces per week) | 1.09 (1.04) | 99.5 | 1.15 (1.09) | 99.5 |
| Non-oily fish (pieces per week) | 1.14 (0.93) | 99.6 | 1.19 (0.94) | 99.6 |
| Red wine intake (glasses per week) | 3.97 (5.82) | 70.1 | 4.03 (6.34) | 83.5 |
| White wine intake (glasses per week) | 2.69 (4.89) | 70.0 | 2.53 (5.70) | 83.6 |
| Beer /cider intake (pints per week) | 3.11 (5.71) | 70.2 | 9.11 (10.26) | 83.8 |
| Spirits intake (measures per week) | 1.98 (5.91) | 70.0 | 2.97 (8.99) | 83.4 |
| Fortified wine intake (glasses per week) | 0.24 (1.31) | 70.2 | 0.22 (1.76) | 83.6 |
| Other alcohol intake (glasses per week) | 0.03 (0.41) | 22.4 | 0.01 (0.20) | 30.2 |
| Coffee consumption, n (%) | 101076 (78.5%) | 99.8 | 1617 (75.7%) | 99.2 |
| Coffee intake (cups per day) | 2.12 (2.20) | 99.8 | 1.75 (1.83) | 99.2 |
| Tea consumption, n (%) | 108734 (84.4%) | 99.8 | 1813 (84.9%) | 99.9 |
| Tea intake (cups per day) | 3.45 (2.99) | 99.8 | 3.31 (2.88) | 99.9 |
| Any fruit consumption, n (%) | 119605 (92.8%) | 98.9 | 1917 (89.8%) | 98.0 |
| Fruit of any description (pieces per day) | 2.98 (2.50) | 98.9 | 2.72 (2.33) | 98.0 |
| Fresh fruit intake (pieces per day) | 2.18 (1.57) | 99.2 | 2.09 (1.55) | 99.2 |
| Dried fruit intake (pieces per day) | 0.80 (1.67) | 99.4 | 0.63 (1.48) | 98.6 |
| Any vegetable consumption, n (%) | 124801 (96.87%) | 98.6 | 2045 (95.8%) | 97.3 |
| All vegetable intake (pieces per day) | 4.83 (3.17) | 98.6 | 4.83 (3.06) | 97.3 |
| Raw vegetable intake (pieces per day) | 2.14 (2.09) | 99.1 | 2.04 (2.10) | 98.1 |
| Cooked vegetable intake (pieces per day) | 2.69 (1.80) | 99.3 | 2.79 (1.66) | 98.3 |
| Any bread consumption, n (%) | 124673 (96.8%) | 99.2 | 2085 (97.7%) | 98.7 |
| Bread intake (slices per week) | 12.52 (8.69) | 99.2 | 14.77 (9.72) | 98.7 |
| Any cereal consumption, n (%) | 111513 (96.6%) | 99.7 | 1744 (81.7%) | 99.9 |
| Cereal intake (bowls per week) | 4.52 (2.80) | 99.7 | 3.83 (2.81) | 99.9 |
| Any cheese consumption, n (%) | 122469 (95.1%) | 97.7 | 1996 (93.5%) | 96.9 |
| Cheese intake (slices per week | 2.46 (1.75) | 97.7 | 2.35 (1.66) | 96.9 |
