# Supplementary material for: Mediation analysis to understand genetic relationships between habitual coffee intake and gout
Source: Arthritis Res Ther. 2018 Jul 5;20:135. doi: 10.1186/s13075-018-1629-5 (PMC6034252; doi:10.1186/s13075-018-1629-5)
Supplement: Supplementary file 5 — Table S3. Association analysis of genotype with gout risk. (DOC 57 kb) [file 13075_2018_1629_MOESM5_ESM.doc]

**Table S3. Association analysis of genotype with gout risk.** Effect allele is allele associated with hyperuricaemia in Kottgen GWAS paper (2).

| **Association of gout with urate-associated SNPs** | | | | | | | | | | | | | | | |
| --- | --- | --- | --- | --- | --- | --- | --- | --- | --- | --- | --- | --- | --- | --- | --- |
| **Gene** | **SNP** | **Effect allele** | **Copies** | **Unadjusted** | | | | **Adjusted†** | | | | **Adjusted† including beer/spirits** | | | |
|  |  |  |  | **Observations** | **Odds ratio** | **95% Confidence Interval** | **P** | **Observations** | **Odds ratio** | **95% Confidence Interval** | **P** | **Observations** | **Odds ratio** | **95% Confidence Interval** | **P** |
| ***GCKR*** | *rs1260326* | T | 0 | 47996 | 1.00 | - | - | 44678 | 1.00 | - | - | 32314 | 1.00 | - | - |
|  |  |  | 1 | 62274 | 1.33 | 1.21 - 1.47 | 1.08E-08 | 57957 | 1.35 | 1.22 - 1.50 | 2.10E-08 | 41017 | 1.37 | 1.22 - 1.54 | 1.19E-07 |
|  |  |  | 2 | 20696 | 1.58 | 1.39 -1.79 | 6.51E-13 | 19305 | 1.65 | 1.44 - 1.88 | 2.01E-13 | 13371 | 1.74 | 1.50 - 2.01 | 2.38E-13 |
| ***ABCG2*** | *rs2231142* | T | 0 | 103321 | 1.00 | - | - | 96159 | 1.00 | - | - | 68388 | 1.00 | - | - |
|  |  |  | 1 | 26015 | 2.15 | 1.96 - 2.36 | 4.59E-60 | 24163 | 2.26 | 2.04 - 2.49 | 6.18E-58 | 17166 | 2.32 | 2.08 - 2.59 | 1.32E-50 |
|  |  |  | 2 | 1730 | 3.94 | 3.15 -4.93 | 5.28E-33 | 1618 | 4.11 | 3.19 - 5.31 | 1.82E-27 | 1148 | 4.59 | 3.47 - 6.06 | 8.36E-27 |
| ***MLIXPL*** | *rs1178977* | A | 0 | 5023 | 1.00 | - | - | 4686 | 1.00 | - | - | 3404 | 1.00 | - | - |
|  |  |  | 1 | 41254 | 1.08 | 0.84 -1.40 | 0.54 | 38376 | 1.08 | 0.83 - 1.42 | 0.56 | 27376 | 1.08 | 0.81 - 1.45 | 0.60 |
|  |  |  | 2 | 84660 | 1.34 | 1.04 - 1.71 | 0.02 | 78852 | 1.31 | 1.01 - 1.70 | 0.04 | 55907 | 1.32 | 0.99 - 1.76 | 0.06 |
| ***CYP1A2*** | *rs2472297* | C | 0 | 9190 | 1.00 | - | - | 8574 | 1.00 | - | - | 6200 | 1.00 | - | - |
|  |  |  | 1 | 50507 | 1.09 | 0.91 - 1.32 | 0.34 | 47013 | 1.10 | 0.90 - 1.34 | 0.34 | 33549 | 1.10 | 0.89 - 1.37 | 0.37 |
|  |  |  | 2 | 71269 | 1.19 | 0.99 -1.42 | 0.07 | 66353 | 1.21 | 0.99 - 1.46 | 0.06 | 46953 | 1.20 | 0.97 - 1.47 | 0.09 |
| ***†Adjusted for age, sex, BMI, hypertension, kidney disease, diabetes, meat intake, fish intake, cheese intake, tea intake, fruit intake, vegetable intake, bread intake, and cereal intake.*** | | | | | | | | | | | | | | | |
