# Supplementary material for: Mediation analysis to understand genetic relationships between habitual coffee intake and gout
Source: Arthritis Res Ther. 2018 Jul 5;20:135. doi: 10.1186/s13075-018-1629-5 (PMC6034252; doi:10.1186/s13075-018-1629-5)
Supplement: Supplementary file 6 — Table S4. Association analysis of genotype with habitual coffee intake. (DOC 54 kb) [file 13075_2018_1629_MOESM6_ESM.doc]

| **Association of coffee intake (cups per day) with urate-associated SNPs** | | | | | | | | | | | | | | | |
| --- | --- | --- | --- | --- | --- | --- | --- | --- | --- | --- | --- | --- | --- | --- | --- |
| **Gene** | **SNP** | **Effect allele** | **Copies** | **Unadjusted** | | | | **Adjusted†** | | | | **Adjusted† including beer/spirits** | | | |
|  |  |  |  | **Observations** | **Unadjusted β-coefficient** | **95% Confidence Interval** | **P** | **Observations** | **Unadjusted β-coefficient** | **95% Confidence Interval** | **P** | **Observations** | **Unadjusted β-coefficient** | **95% Confidence Interval** | **P** |
| ***GCKR*** | *rs1260326* | T | 0 | 47996 | - | - | - | 44669 | - | - | - | 32304 | - | - | - |
|  |  |  | 1 | 62274 | -0.06 | -0.09 - -0.04 | 4.00E-06 | 57939 | -0.06 | -0.09 - -0.04 | 8.94E-07 | 41005 | -0.06 | -0.09 - -0.03 | 3.60E-05 |
|  |  |  | 2 | 20696 | -0.10 | -0.14 - -0.07 | 2.10E-08 | 19299 | -0.12 | -0.15 - -0.08 | 3.86E-11 | 13367 | -0.12 | -0.16 - -0.09 | 2.44E-10 |
| ***ABCG2*** | *rs2231142* | T | 0 | 103221 | - | - | - | 96124 | - | - | - | 68367 | - | - | - |
|  |  |  | 1 | 26015 | -0.07 | -0.10 - -0.04 | 7.00E-06 | 24156 | -0.08 | -0.11 - -0.05 | 6.37E-08 | 17162 | -0.07 | -0.10 - -0.04 | 1.50E-05 |
|  |  |  | 2 | 1730 | -0.12 | -0.22 - -0.01 | 2.80E-02 | 1617 | -0.18 | -0.28 - -0.08 | 4.32E-04 | 1147 | -0.23 | -0.34 - -0.12 | 4.70E-05 |
| ***MLIXPL*** | *rs1178977* | A | 0 | 5023 | - | - | - | 4683 | - | - | - | 3402 | - | - | - |
|  |  |  | 1 | 41254 | -0.13 | -0.19 - -0.06 | 9.30E-05 | 38368 | -0.14 | -0.20 - -0.07 | 1.70E-05 | 27372 | -0.18 | -0.24 - -0.11 | 3.93E-07 |
|  |  |  | 2 | 84660 | -0.16 | -0.22 - -0.09 | 9.27E-07 | 78820 | -0.18 | -0.24 - -0.12 | 2.04E-09 | 55887 | -0.22 | -0.29 - -0.16 | 3.91E-11 |
| ***CYP1A2*** | *rs2472297* | C | 0 | 9190 | - | - | - | 8571 | - | - | - | 6199 | - | - | - |
|  |  |  | 1 | 50507 | -0.16 | -0.21 - -0.11 | 6.65E-11 | 46996 | -0.19 | -0.24 - -0.14 | 3.32E-15 | 33538 | -0.18 | -0.24 - -0.13 | 3.92E-12 |
|  |  |  | 2 | 71269 | -0.31 | -0.36 - -0.27 | 2.34E-38 | 66330 | -0.38 | -0.43 - -0.34 | 3.80E-60 | 46939 | -0.38 | -0.43 - -0.33 | 3.17E-49 |
| ***†Adjusted for age, sex, BMI, hypertension, kidney disease, diabetes, meat intake, fish intake, cheese intake, tea intake, fruit intake, vegetable intake, bread intake, and cereal intake.*** | | | | | | | | | | | | | | | |

**Table S4**. **Association analysis of genotype with habitual coffee intake.** Effect allele is allele associated with hyperuricaemia in Kottgen GWAS paper (2).
